# The Insulin-Like Proteins dILPs-2/5 Determine Diapause Inducibility in *Drosophila*

**DOI:** 10.1371/journal.pone.0163680

**Published:** 2016-09-30

**Authors:** Luca Schiesari, Gabriele Andreatta, Charalambos P. Kyriacou, Michael B. O’Connor, Rodolfo Costa

**Affiliations:** 1 Department of Biology, University of Padova, Padova, Italy; 2 Department of Genetics, Cell Biology and Development, University of Minnesota, Minneapolis, United States of America; 3 Department of Genetics, University of Leicester, Leicester, United Kingdom; University of Mississippi, UNITED STATES

## Abstract

Diapause is an actively induced dormancy that has evolved in Metazoa to resist environmental stresses. In temperate regions, many diapausing insects overwinter at low temperatures by blocking embryonic, larval or adult development. Despite its Afro-tropical origin, *Drosophila melanogaster* migrated to temperate regions of Asia and Europe where females overwinter as adults by arresting gonadal development (reproductive diapause) at temperatures <13°C. Recent work in *D*. *melanogaster* has implicated the developmental hormones dILPs-2 and/or dILP3, and dILP5, homologues of vertebrate insulin/insulin-like growth factors (IGFs), in reproductive arrest. However, polymorphisms in *timeless (tim)* and *couch potato (cpo)* dramatically affect diapause inducibility and these dILP experiments could not exclude this common genetic variation contributing to the diapause phenotype. Here, we apply an extensive genetic dissection of the insulin signaling pathway which allows us to see both enhancements and reductions in egg development that are independent of *tim* and *cpo* variations. We show that a number of manipulations dramatically enhance diapause to ~100%. These include ablating, or reducing the excitability of the insulin-producing cells (IPCs) that express dILPs-2,3,5 employing the *dilp2*,*3*,*5*^*-/-*^ triple mutant, desensitizing insulin signaling using a *chico* mutation, or inhibiting dILP2 and 5 in the hemolymph by over-expressing Imaginal Morphogenesis Protein-Late 2 (Imp-L2). In addition, triple mutant *dilp2*,*3*,*5*^-/-^ females maintain high levels of diapause even when temperatures are raised in adulthood to 19°C. However at 22°C, these females all show egg development revealing that the effects are conditional on temperature and not a general female sterility. In contrast, over-expression of *dilps-2/5* or enhancing IPC excitability, led to levels of ovarian arrest that approached zero, underscoring dILPs-2 and 5 as key antagonists of diapause.

## Introduction

In temperate regions, several holometabolous insects overwinter in a state of diapause, an actively induced dormancy that blocks developmental growth at species-specific stages of their life cycles and enhances cold tolerance [1]. *Drosophila melanogaster*, despite its Afro-tropical origins, has evolved a diapause response in temperate regions where adult females induce reproductive dormancy by arresting gonadal growth in response to cold environmental conditions below 13°C in combination with photoperiodic stimuli [2–4]. A fine-tuned regulation of diapause timing is important for synchronizing *Drosophila*’s life-cycle with environmental changes and several genes have been implicated in reproductive arrest under colder temperatures and shorter photoperiods.

The *s-tim/ls-tim* natural polymorphism in the circadian clock gene *timeless* generates a substantial difference in diapause inducibility as revealed in several natural populations and confirmed with transgenic lines, where the recently derived *ls-tim* mutation enhances diapause at all photoperiods [2,5]. Two closely linked polymorphisms in *couch potato (cpo)*, *cpo*^*A356V*^ and *cpo*^*I462K*^ (now reannotated as *cpo*^*A347V*^ and as intronic polymorphism SNP 48034(A/T)–[6]) show latitudinal clines in North America, have also been suggested to modulate diapause induction [7] but no relationship between these polymorphisms and diapause is present in Australian populations [8]. *cpo* lies between the proximal and distal breakpoints of the cosmopolitan *Payne* inversion, *In(3R)P*, which shows a cline in frequency on both continents complicating interpretation of the *cpo* cline [9,10]. Nevertheless, complementation analysis reveals that these two natural *cpo* variants as well as artificially generated mutations of *cpo*, significantly affect diapause with higher levels of diapause inversely correlated with *cpo* expression levels [7].

In addition, natural variation in another gene that lies within *In(3R)P* and which encodes insulin-regulated phosphatidylinositol 3 kinase (PI3K), *dp110*, appears to affect diapause levels and may also contribute to a latitudinal cline in diapause induction that is observed in North America, but the molecular basis for the polymorphism remains obscure [11,12]. The *insulin-like receptor (InR)* gene also lies within *In(3R)P* and encodes considerable polymorphism, with one allele showing a similar latitudinal cline in frequency in both North America (standard chromosome only) and Australia (inverted chromosome only) [13]. Heteroallelic mutational combinations in *InR* have long been known to generate female sterility as the eggs remain previtellogenic at 25°C but whether these polymorphisms affect diapause in *Drosophila* has yet to be studied [14,15]. Thus, until recently, there was only circumstantial evidence that insulin signaling might be involved in conditional ovarian developmental arrest at low temperatures in *D*. *melanogaster*.

However, in a recent study of *D*. *melanogaster* diapause, females maintained in diapause for several weeks showed elevated gene expression for *Drosophila* insulin-like peptides, dILPs 2–6 [16]. If there exists a causal relationship between diapause and *dilp* levels, then mutations in some of these *dilp* genes might be expected to reduce levels of diapause. In contrast, mutations in *dilp2-3* and *dilp5* appeared to enhance the depth of diapause as measured by ovarian development [16], possibly caused by compensatory up-regulation of other dILPs [17]. One potential problem with this experiment, apart from the small numbers of female flies that were analysed, was that the wild-type and *dilp2-3*^*-/ -*^and *dilp5*^*-/-*^ mutants were not assessed for either their *cpo* or *tim* genotypes which could conceivably have generated their different diapause profiles.

The *Drosophila* genome encodes a number of *insulin/insulin-like protein* genes (*dilps)* [18–21]. Two of them, *dilps-2/5*, are mainly expressed in a cluster of Median Neurosecretory Cells (MNCs), the insulin-producing cells (IPCs) [18,19]. *dilps-2/5* are expressed independently in IPCs and differentially during normal development with *dilp2* showing an earlier and stronger larval expression than *dilp5* [18,22]. *dilps-2/5* encode small peptide hormones of 137 aa and 107 aa, respectively, which are released into the haemolymph and signal through InR to inhibit the transcription factor Forkhead box-O (FoxO) in target organs. dILP-2/5 act redundantly to control a plethora of developmental and physiological functions such as larval growth rate, metamorphic timing, energy metabolism, fecundity and aging [17–26].

Given the available evidence that suggests insulin signaling involvement in the *Drosophila* overwintering response, we sought to further dissect the role of these *dilps* in diapause induction by using an extensive set of genetic manipulations on known *tim* and *cpo* genetic backgrounds to clarify the role of insulin signaling in this important seasonal adaptation.

## Results/Discussion

We screened all our lines for the *tim* [2] and *cpo* [7] natural alleles (see Materials and Methods). Among our lines, the single *dilp2*^*-/-*^, *dilp5*^*-/-*^, *dilp3*^*-/-*^, *chico*^*KG00032*^ mutants, *UAS-dilp2 RNAi*, *UAS-dilp5RNAi*, *UAS-sNPF;UAS-sNPF*, *akh-Gal4* and *Lk6*^*DJ634*^*-Gal4* carry the *ls-tim* allele so we used a corresponding *ls-tim* as a control. For *cpo*, the upstream *C/T* (*cpo*^*A347V*^) substitution showed some variation among strains whereas the downstream SNP *48034(A/T)* substitution was represented as *A* in all strains except for *UAS-hid*,*rpr* which carried a *T* and is reported to promote reduced levels of diapause [6,7]. S1 Table carries the summary of all the diapause results and the background genotypes of all strains tested.

### Manipulation of IPCs

In order to test whether neural dILPs-2,3,5 play a key role in diapause induction, we removed dILPs-2,3,5 signaling late in the larval stage (from mid-late third larval instar, L3) by ablating the IPCs, the main source of these dILPs [18,19], using the *Gal4/UAS* system. We used two different drivers, *dilp2-Gal4* (*dilp2>*) and *InsP3-Gal4* (*InsP3>*), which express Gal4 from mid-late L3 [17,27] and late L3/pupa [28], respectively, in order to drive the expression of two pro-apoptotic genes simultaneously, *hid* (*head involution defective*) and *rpr* (*reaper*) in IPCs. Because of their later larval expression, these drivers do not cause the lethality or severe developmental defects [27,28] that are induced by the early larval ablation of IPCs with the precocious *dilp2(p)*-*Gal4* (*dilp2(p)>*) driver [18].

**Fig 1:**
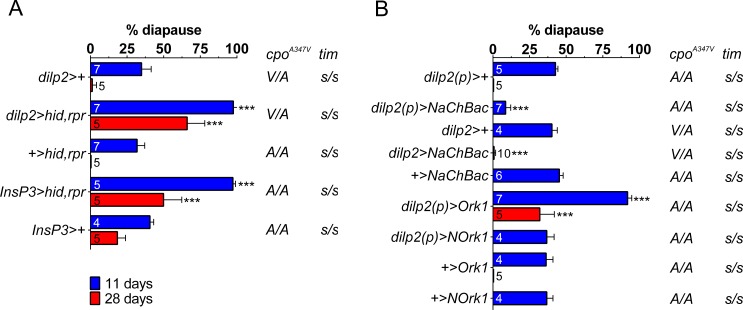
Insulin-producing cells (IPCs) regulate reproductive dormancy in *D*. *melanogaster*. (A) Ablation of IPCs with *dilp2>hid*,*rpr* and *Insp3>hid*,*rpr* significantly enhances diapause at both 11 and 28 days compared with controls even under a long summer photoperiod (LD16:8). (B) Hyperexcitability of IPCs decreases diapause frequency (*NaChBac*) whereas reducing excitability (*Ork1)* significantly enhances diapause also compared to the non-conducting control *NOrk*1. Blue 11 days, red 28 days. Numbers within bars represent replicates (see Methods). Mean ± SD, ***p<0.001. (ANOVA was performed using the arcsin transformation but the results are presented as percentages for simplicity).

Strikingly, IPCs ablation (*dilp2>hid*,*rpr* and *Insp3>hid*,*rpr*) promotes a near complete diapause response (97.6 ± 2.9% and 97.3 ± 1.7%, respectively) compared to controls (Fig 1A).

This is not female sterility because at 23°C these females all produce late stage eggs (S2 Table and S1 Fig, also shown by others [29]). If the ablations were simply causing a slowing down in maturation of the gonads rather than an arrest, we should observe that after a longer period in diapause inducing conditions, the experimental genotypes should ‘catch up’ and give similar levels of diapause to controls. We therefore maintained our females for 28 days at 12°C and observed that diapause levels fell to 0–1% in *dilp2>+* and *UAS-hid*,*rpr* controls, and 18% in *InsP3>+* but were maintained at 66% and 50% in *dilp2>hid*,*rpr* and *InsP3>hid*,*rpr* respectively (Fig 1A). These results reveal a key role for neural IPCs and their functions in modulating and maintaining *Drosophila* diapause at colder temperatures.

To further understand the role of IPCs on reproductive dormancy, we disrupted the neuronal physiology of IPCs (and, in turn, the release of dILPs) by over-expressing a potassium (*UAS-dOrkΔ-C*, designated as *UAS-Ork1*) or a sodium channel (*UAS-NaChBac*) which reduce or enhance neuronal excitability, respectively [30,31]. *Ork1ΔC* over-expression from early larval life (*dilp2(p)>Ork1*) promotes very high levels of reproductive diapause in a similar manner to IPCs ablation (91.9 ± 2.8%) compared to its *dilp2(p)>dORKΔ-NC (NOrk1)* non-conducting control (36.8 ± 5.0%) whereas *NaChBac* over-expression (*dilp2(p)>NaChBac*) inhibits ovarian dormancy and induces gonadal growth in the cold (Fig 1B), reinforcing the notion that dILPs have a regulatory role on dormancy. Again *Ork1ΔC* over-expression females produced stage 14 eggs at 23°C and after 28 days, *dilp2(p)>Ork1* females still showed >30% diapause compared to their corresponding driver and *UAS* controls that showed almost zero diapause (S1 Table and Fig 1B).

The higher levels of diapause in the experimental flies illustrated in Fig 1 are generated even though these females carry the low diapause *s-tim* allele and were maintained in the longer summer LD16:8 photoperiod. Furthermore, the most frequent *cpo* genotype in these lines was *C/C A/A* (*cpo*^*347Ala*^, *cpo*^*48034A*^, S1 Table). Zonato et al (submitted) reveals that the *cpo*^*48034A/T*^ polymorphism does not play any role in diapause at either 12 or 28 days in the *s-tim* background. However the *cpo*^*A347V*^ polymorphism was observed to have a significant effect with *cpo*^*Val*^ showing higher levels of diapause than *cpo*^*Ala*^. S1 Table shows the relevant *cpo*^*A347V*^ genotypes for all the lines, and some are heterozygous, while the majority are homozygous for *cpo*^*Ala*^. If we compare the control strains we observe at 11 and 28 days, diapause levels lie between 32–47% and 0–18% respectively, but there is no correlation with *cpo*^*A347V*^ genotype, suggesting that the heterozygous combination of alleles does not enhance diapause compared with *cpo*^*Ala*^ homozygotes. Consequently, *cpo*^*A347V*^ genotypes in the *s-tim* background do not appear to play any significant role in the large changes we observe in diapause induction compared to the dramatic effects we observe when we manipulate the IPCs.

### The effects of altered expression of *dilps* on diapause induction

**Fig 2:**
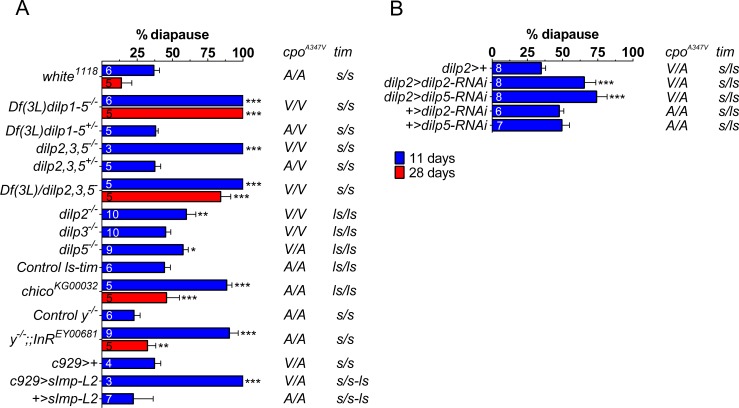
*dilps-2/5* redundantly inhibit diapause. (A) Combined *dilps-2/5* null mutations promote diapause induction (*Df(3L)dilp1-5*^*-/-*^ and *dilp2*,*3*,*5*^*-/-*^) at both 11 and 28 days. Among three *dilps* expressed in IPCs, only *dilp2*^*-/-*^ and *dilp5*^*-/-*^ single mutations modestly enhance diapause frequency. Hypomorphic mutations in *chico* and *InR* also significantly enhance diapause as does the over-expression of *sImp-L2* using the neuropeptide cell driver *c929*. (B) Similarly RNAi driven by *dilp2* promoter significantly enhances diapause induction. Mean ± SD, ANOVA on arcsin transformations, *p<0.05, **p<0.01, ***p<0.001.

To investigate whether it was the dILPs within the IPCs that were generating these effects, we also examined whether a chromosomal deficiency uncovering five of the eight *dilp*s, *Df(3L)dilp1-5*^*-/-*^ [25], disrupted the wild-type response. Strikingly, *Df(3L)dilp1-5*^*-/-*^ mutants induce 100% diapause at 12°C which is maintained at this level for 28 days whereas both the heterozygous mutant (*Df(3L)dilp1-5*^+/-^) and matched background controls (*w*^*1118*^) exhibited 38.1 ± 1.9% and 36.8 ± 4.0% diapause respectively (Fig 2A).

Similarly, the two genotypes that were triply mutant, *dilp2*,*3*,*5*^*-/-*^ [17] and *Df(3L)dilp1-5/dilp2*,*3*,*5*^*-*^ induced maximum diapause which in the latter genotype was maintained for 28 days at 84% (Fig 2A).

We also investigated hypomorphic *chico* mutants (*chico*^*KG00032*^) [23], that disrupt dILPs sensitivity, and observed that they too exhibited very high levels of diapause (89% falling to 46% after 28 days, Fig 2A) Similarly the weakly hypomorphic *InR*^*EY00681*^ mutant [32] also showed very high levels of diapause (90.3 ± 6.2%) after 11 days, and over-expressing *Imaginal Morphogenesis Protein—Late 2* (*Imp-L2*), using the neuroendocrine cell driver *c929>* [33,34], provoked 100% diapause, consistent with the function of Imp-L2 in inhibiting dILPs in the haemolymph [35]. All of these experiments were carried out at LD16:8 and all experimental genotypes carried the *s-tim* background that favors low diapause levels (except *Imp-L2*, where *s/ls* were segregating, Fig 2A). In contrast, none of the single null mutants (*dilp2*^*-/-*^, *dilp3*^*-/-*^ or *dilp5*^*-/-*^) caused strong induction of diapause, compared to *Df(3L)dilp1-5*^*-/-*^, although individually, both *dilp2*^*-/-*^ and *dilp5*^*-/-*^ modestly enhanced the frequency of diapause (59.8 ± 7.0% and 57.5 ± 3.7%, respectively, Fig 2A) compared to controls sharing the same *ls-tim* backgrounds. As *dilp3*^*-/-*^ was homozygous for the diapause promoting *cpo*^*Val*^ allele, whereas *dilp5*^*-/-*^ was polymorphic at this site, any enhancement of diapause in the latter compared to *dilp3*^*-/-*^ is possibly underestimated. These results, coupled with the suggested autocrine regulatory role of dILP3 [17] lead us to suggest dILP2 and 5 as the key IPCs-released dILPs for diapause suppression. A similarly modest but significant enhancement was observed on knocking down *dilp2* (65.5 ± 8.1%) or *dilp5* (74.2 ± 7.4%) with RNAi using the *dilp2* promoter, compared to the corresponding *UAS-RNAi* controls which both gave just under 50% diapause (Fig 2B). The knocked down flies were heterozygous for *cpo*^*Val*^ whereas the controls were homozygous for *cpo*^*Ala*^, which could possibly explain why these effects were a little more dramatic than the single mutants (S1 Table).

Both *Df(3L)dilp1-5*^*-/-*^ and *dilp2*,*3*,*5*^*-/-*^ nulls produce dwarf adults and developmentally delayed larvae [17,25], suggesting that dILPs-2/5 control of diapause is pleiotropically linked to larval growth. Yet although *Df(3L)dilp1-5*^*-/-*^ and single null mutants (*dilp2*^*-/-*^, *dilp3*^*-/-*^ or *dilp5*^*-/-*^) or late IPCs ablation (*dilp2>hid*, *rpr*) exhibit reduced fecundity [17,19,25], these females all lay viable embryos at 23°C whereas at 12°C oogenesis is blocked. In addition, the single *dilp3*^*-/-*^ mutation causes fecundity defects [17], but it does not promote an enhanced diapause response revealing a decoupling of diapause from sterility. Furthermore, in our hands, the experimental genotype females are vitellogenic at 23°C (S2 Table and S1 Fig). Consequently *dilps-2/5* genes appear to redundantly and specifically control a conditional temperature-dependent diapause/development switch.

**Fig 3:**
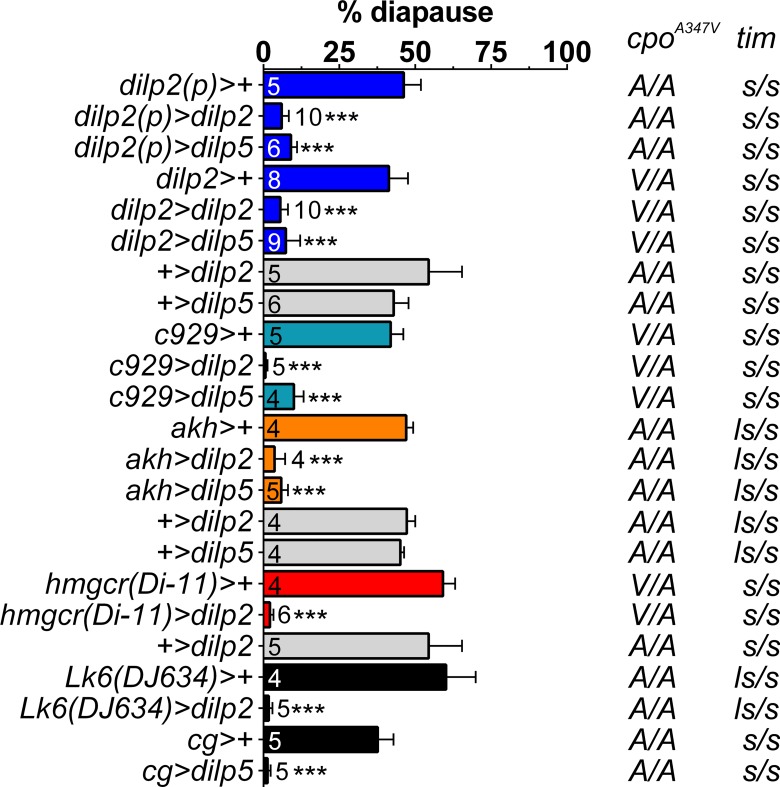
Over-expression of *dilp2* and *dilp5* dramatically reduces diapause levels. *dilps-2/5* over-expression from early (*dilp2(p)>dilp2* and *dilp2(p)>dilp5*) or late (*dilp2>dilp2* and *dilp2>dilp5*) larval stages inhibit diapause at 12°C under short photoperiod (LD8:16). Mean ± SD, ANOVA on arcsin transformations, ***p<0.001. The same result is observed with ectopic drivers (see text for details).

If combined *dilps-2/5* loss, reducing sensitivity or inhibiting dILPs-2/5 promotes diapause under cold conditions, then conversely, *dilps-2/5* over-expression might abolish the ability to induce diapause. We therefore over-expressed *UAS-dilp5* or *UAS-dilp2* transgenes from early L2 (*dilp2(p)>dilp5* and *dilp2(p)>dilp2*) or mid-late L3 instar (*dilp2>dilp5* and *dilp2>dilp2*). Strikingly, these manipulations caused almost complete inhibition of reproductive diapause at 12°C (even though these females were maintained in diapause-promoting winter LD8:16 photoperiods Fig 3).

Furthermore, we also induced ectopic *dilps-2/5* over-expression with a number of promoters for neuroendocrine and endocrine cells (*c929*, [33,34]), larval (cg, [22]) or adult (*Lk6*^*DJ634*^, [36,37]), fat-body, *corpus allatum* (*hmgcr*^*Di-11*^ [38,39]) and *corpus cardium* (*akh*) [40]. All of these manipulations significantly reduced diapause inducibility in a similar manner to dILPs-2/5 over-expression in MNCs (Fig 3), suggesting that irrespective of the tissue from which dILPs are expressed and the timing of their release, it is their presence in the hemolymph that is the critical factor. Taken together, these results suggest that dILPs-2/5 are the key antagonists of diapause and that they lie at the core of the genetic mechanism underlying induction of ovarian dormancy.

**Fig 4:**
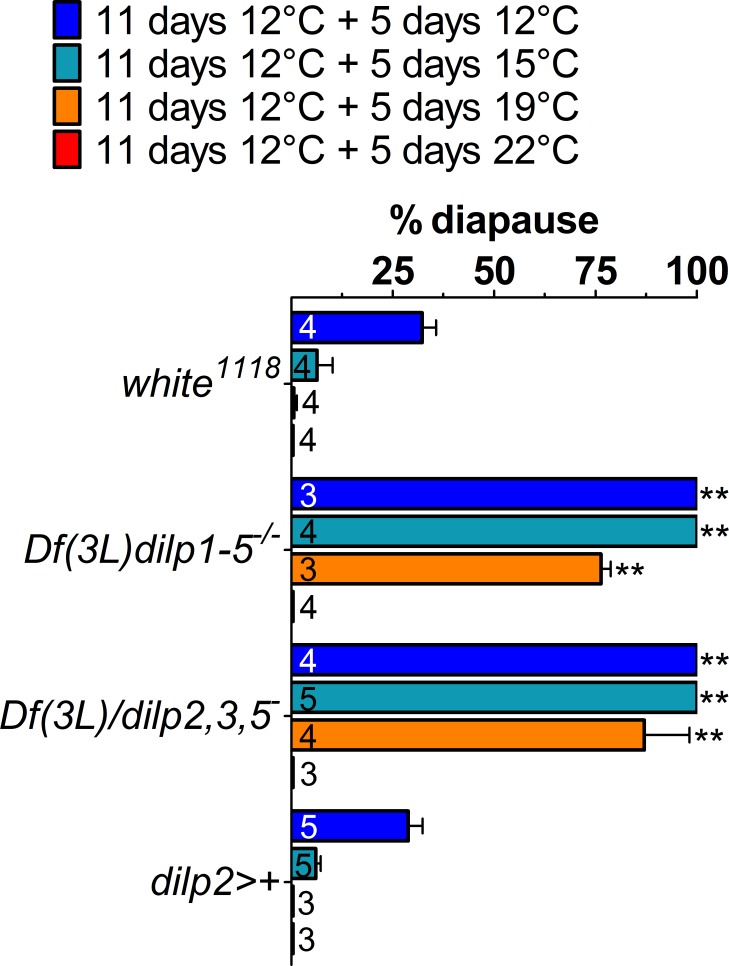
Combinations of *dilp* mutations maintain diapause under more favourable conditions. Under warmer temperatures and longer photoperiods (LD16:8), conditions favourable to growth, *Df(3L)dilp1-5*^*-/-*^ and *Df(3L)dilp1-5/dilp2*,*3*,*5*^*-*^ mutants remain in diapause at high levels even at 19°C compared to controls, but ovaries mature at 23°C. Mean ± SD, ANOVA on arcsin transformations, **p<0.01.

Wild-type diapausing flies can switch from diapause to full fecundity if they re-encounter a favorable environment [16]. If dILPs-2/5 levels are critical for modulating the diapause response, then we expect that *Df(3L)dilps1-5*^*-/-*^ mutants should not transition out of diapause as quickly if they experience a shift to favorable conditions. We therefore exposed *Df(3L)dilps1-5*^*-/-*^ and *Df(3L)dilp1-5/dilp2*,*3*,*5*^*-*^ null mutants to diapause-inducing conditions (12°C) for 11 days before switching them for an additional 5 days to non-diapausing conditions (15°C, 19°C, or 22°C). We observed that even at 19°C, the null mutants maintained high frequencies of diapause compared to the controls (Time x Genotype F_6,42_, 18.2 *p*<0.01, Fig 4).

Only at 23°C did the nulls start to mature their gonads, suggesting that dILPs are the limiting signals determining diapause in colder conditions. These results are important as they show clearly that these mutants are conditional sterile at low temperature and reinforce the view that we are examining diapause, a reversible phenomenon, not simply female sterility.

**Fig 5:**
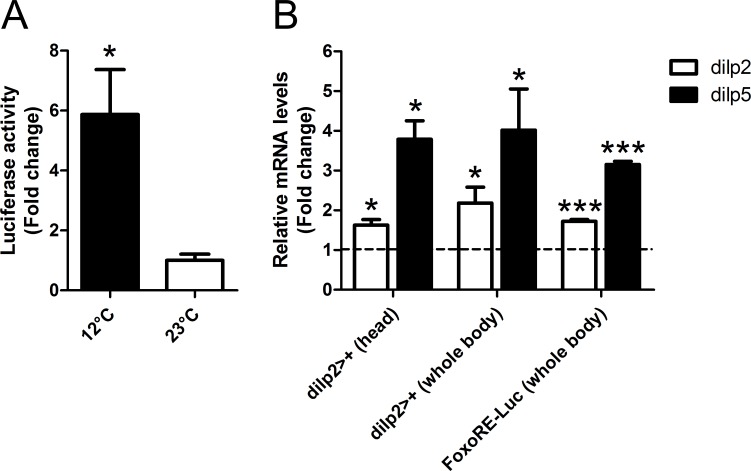
Regulation of *FoxO* and *dilp2/5* in diapausing females. (A) *FoxO*.*RE-Luciferase* reporter gene assay. Reporter activity in diapausing (12°C) *versus* non-diapausing flies (23°C) is shown (p = 0.032, t-test). Reporter activity is higher in flies abdomen at 12°C. Y-axis: Luciferase activity (fold change). (B) qPCR of *dilps-2/5* mRNA levels in two different ‘wild-type’ genotypes, *FoxO*.*RE*.*Luciferase* used in A and *dilp2>+*. *dilps-2/5* expression levels of diapausing (12°C) *versus* non-diapausing flies (23°C) are shown (ratio: diapausing/non-diapausing). *dilps-2/5* are up-regulated in diapausing flies. Y-axis: mRNA levels (fold change). Dotted line indicates the expression levels in non-diapausing flies. Mean ± SE, *p<0.05, ***p<0.001 are based on t-test.

If our proposal is correct, then dILPs-2/5 signaling should be perturbed by environmental conditions that induce reproductive diapause. In particular, we predict that if dILPs-2/5 inhibit reproductive diapause, then dILPs-2/5 signaling should be repressed during dormancy induction. To address this issue, we used a FoxO response element-luciferase reporter (*FoxO*.*RE-Luc)* [41] to estimate the levels of insulin/FoxO signaling in diapausing flies (reared at 12°C, LD8:16) *versus* “non-diapausing” ones (reared at 23°C). Under conditions of reduced insulin signaling FoxO should enter the nucleus and up-regulate luciferase activity. Consistent with the notion that reduction in dILPs-2/5 signaling promotes reproductive dormancy, diapausing flies exhibited a significant (p<0.05) 6-fold up-regulation of FoxO activity in isolated abdomen (containing the gonads, direct downstream targets of brain dILPs [42]) (Fig 5A).

In other cases of insulin signaling down-regulation, there is often an accompanying compensatory enhancement of *dilps-2/5* gene transcription in the IPCs. For example, increased mRNA levels of *dilps-2/5* are observed in a *SH2B*^*-/-*^ mutant (SH2B is an intracellular adaptor of InR) [23] or when *Imp-L2* is over-expressed [43]. In *dilp2*^*-/-*^ mutants there is up-regulation of the *dilp3* and *dilp5* transcripts, while in *dilp5*^*-/-*^ mutants, *dilp3* is up-regulated, with *dilp2-3*^*-/-*^ double mutant also enhancing *dilp5* expression [17]. As *dilp3*^*-/-*^ mutants also reduce *dilp2* and *dilp5* expression, there is both positive and negative cross-regulation among these *dilps* [17]. Similar up-regulation of *dilps-2/5* transcripts have also been reported upon *Imp-L2* over-expression [43] and ablation of the germ line [44] which are direct targets of brain dILPs [42]. Thus, paradoxically, we predicted that diapausing flies might exhibit up-regulation of *dilps-2/5* genes as a compensatory response. Strikingly, the expression levels of *dilps-2/5* genes were both up-regulated in reproductively diapausing flies (reared at 12°C) *versus* non-diapausing flies (reared at 23°C), in both whole body and head-only samples of two different ‘wild-type’ strains, *dilp2>+* and *FoxO*.*RE-Luciferase* (Fig 5B), both of which showed normal levels of diapause (40.3 ± 3.7% and 49.5 ± 4.8% in LD8:16 and 33.2 ± 4.6% and 36.2 ± 3.4% in LD16:8, respectively). A similar result was obtained with diapausing Canton-S flies by Kubrak et al [16]. Although this feedback mechanism remains to be clarified, these results support the notion that dILPs-2/5 signaling fails under the perturbing effects of an adverse environment and this is the key event for diapause induction.

We note that the up-regulation of *dilps-2/5* mRNA levels is the opposite to that reported in the monarch butterfly [45] and mosquito [46] where diapause correlates with down-regulation of *insulin/IGFs* expression. This is a curious difference, but variation in hormonal regulation among insect species, especially with respect to diapause or metamorphosis regulation, is quite common (reviewed in [1]). For example, Juvenile hormone prevents metamorphosis by specifying larval molts in Lepidoptera but it does not in *Drosophila*, [47,48]). Independently of the feedback mechanism that regulates the compensatory phenotype, the key aspect of our results is that, in *Drosophila*, reproductively diapausing flies exhibit a disruption of the normal systemic dILPs-2/5 signaling, which is consistent with the shutdown of this hormonal signaling pathway during diapause of other insect species [1,45,46] and the role of insulin-like peptides as diapause antagonists.

Our findings provide direct genetic evidence that dILPs-2/5 signaling is a central regulator of reproductive diapause in *Drosophila* that is independent of *tim* and *cpo* genetic backgrounds and supports and significantly extends the work on *dilp2-3*^*-/-*^ and *dilp5*^*-/-*^ mutants of Kubrak et al [16]. Our primary data demonstrate that loss of dILPs-2/5 signaling promotes, whereas the over-expression of *dilps-2/5* prevents ovarian dormancy. The level of change between these two genetic alternatives (~100%) far outstrips any changes in diapause levels that are determined by *s-tim/ls-tim* or by variants in *cpo*. We can now reconsider the results of Kubrak et al [16] who observed significantly reduced levels of ovarian development in *dilp5*^*-/-*^ and *dilp2-3*^*-/-*^ double mutants. The former single mutant is in a diapause promoting *ls-tim* background and has a polymorphic *cpo*^*A347V*^ genotype so the effects may have been over-estimated by Kubrak et al [16], depending on whatever were the background genotypes of their controls. In a controlled genetic background, any effect on diapause of the single *dilp5*^*-/-*^ or *dilp2*^*-/-*^ mutants is modest in our hands while *dilp3*^*-/-*^ has no effect. However, combining *dilps2*,*3*,*5* mutations generates 100% diapause, demonstrating the epistatic nature of these mutations which will largely bypass the mRNA compensation phenotypes observed in each of the single mutants by Grönke et al [17].

**Fig 6:**
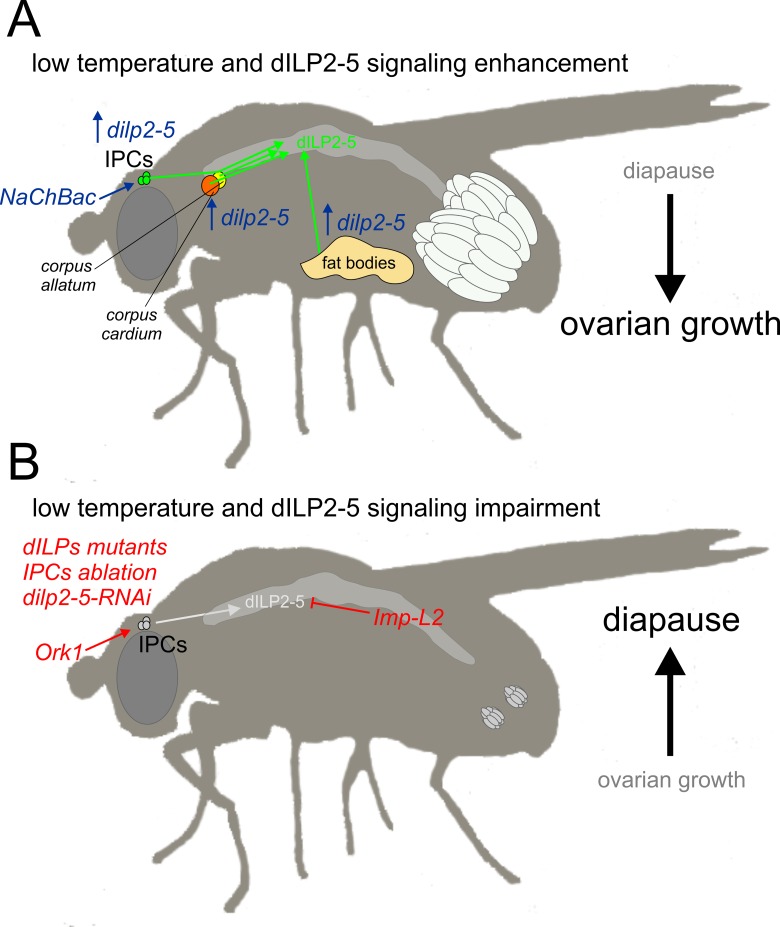
Schematic summary of dILP2-5 signaling effects on diapause. (A) dILP2-5 signaling enhancement, obtained either through IPCs sensitization (NaChBac expression) or *dilp2-5* over-expression (within IPCs or in other endocrine tissues, corpus allatum, corpus cardium and fat bodies) propels ovarian development even under diapause-inducing conditions (low temperature, 12°C, and short photoperiod). (B) Impairment of dILP2-5 signaling through Ork1 expression within IPCs, insulin signaling mutants (both *dilp*, *InR* and *chico* mutants), IPCs ablation, *dilp2-5*-RNAi, or over-expressing the dILPs inhibitor Imp-L2, consolidates diapause preventing further ovarian maturation.

It is further worth pointing out that the changes we have observed by manipulating dILPs signaling are conditional on temperature, so, for example, *dilps2-3*,*5*^*-/-*^ null mutants are not sterile, even though they remain in ovarian arrest at temperatures up to 19°C. Indeed all the variants we have used are fertile at 23°C, even the most severe, *Df(3L)dilp1-5*^-/-^, which as a homozygote has about 10% fertility and 50% viability at this temperature [25]. At 12°C this variant showed 100% diapause, so even partially fertile females show a temperature-sensitive phenotype. Furthermore, by extending some of our key observations to 28 days we avoid basing our interpretations solely on the first two weeks of diapause which can show some fluctuation in the dynamics of egg development [16]. We conclude that our extensive genetic manipulations of insulin-like signaling in *D*. *melanogaster* reveal dILPs-2/5 to be the key regulators and antagonists of seasonal diapause. Fig 6 shows a schematic summary of our major findings.

## Materials and Methods

### Fly Stock and Maintenance

Flies were reared at 23°C under LD12:12 in cornmeal standard food. The following lines were used in this study: *dilp2-Gal4*, *UAS-sImp-L2* and *UAS-dilp2-RNAi* were gifts from Linda Partridge; *dilp2(p)-Gal4* (p, precocious) was a gift from Eric J. Rulifson; *UAS-dilp2* and *UAS-dilp5* were gifts from Ernst Hafen; *InsP3-Gal4* was a gift from Michael J. Pankratz; *Df(3L)dilp1-5*^*-/-*^ was a gift from Leslie Pick; *FoxO*.*RE-Luciferase* was a gift from Brian Staveley; *c929-Gal4*, *UAS-NaChBac*, *UAS-dOrkΔ-C* (designated as *UAS-Ork1*) and its negative control *UAS-dOrkΔ-NC* (designated as *UAS-NOrk1*) were gifts from Michael B. O’Connor; *hmgcr*^*Di-11*^*-Gal4* was a gift from Jean-René Martin, *UAS-hid*,*rpr* was a gift from John R. Nambu; *white*^*1118*^ *s-tim* was a gift from Charlotte Helfrich-Förster. We used a (red-eyed) *white* mutant carrying (but not expressing) *UAS-sNPF/+;UAS-sNPF/+* (parental line *UAS-sNPF;UAS-sNPF* was a gift from Kweon Yu) as the control for the single *dilp* mutants (also in a white background, but with red eyes). *Df(3L)dilp2-3*,*dilp5*^*3*^ (30889), designated in this study as *dilp2*,*3*,*5*^*-/-*^; *dilp5*^*1*^ (30884) designated as *dilp5*^*-/-*^; *dilp2*^*1*^ (30881), designated as *dilp2*^*-/-*^; *dilp3*^*1*^ (30882), designated as *dilp3*^*-/-*^; *chico*^*KG00032*^ (14337); *InR*^*EY00681*^ (15306); *pdf-Gal4* (6900); *akh-Gal4* (25684); *Lk6*^*DJ634*^*-Gal4* (8614); *cg-Gal4* (7011) were from Bloomington *Drosophila* Stock Center; *UAS-dilp5-RNAi* (v49520) was from VDRC; *white ls* from our lab.

### Diapause

Larvae were reared in 12:12 Light/Dark cycles (LD12:12) at 23°C until pupal eclosion. Newly-eclosed adults (~ 60 females and 60 males) were collected within 5 hours of eclosion and exposed to 12°C (diapause-inducing temperature). Each of these samples provided a single ‘replicate’. After 11 days at least 50–60 surviving females were dissected (this number was reduced at 28 days) and scored as “diapausing” or not depending on the complete absence of developing vitellogenic oocytes in both gonads, in accordance with references [2, 7]. This provides a reliable and unambiguous all or none readout of the phenotype [7]. ANOVA (with Tukey *post-hoc* tests) were performed using R statistical software (2.15.1) on the arcsin transformed diapause percentages.

### Genotyping of *timeless* locus

Amplification Refractory Mutation System (ARMS) PCRs [2] was performed to identify *tim* alleles. Genomic DNA was extracted independently from 10 males of each strain. Their homogenate was incubated at 37°C for 45 min in 50μL Solution A (Tris HCl pH8.2 10mM, EDTA 2mM, NaCl 25mM) plus 1μL Proteinase K (10mg/mL) and, then, 3 min at 100°C. Supernatant was processed via ARMS PCR as in [2]. Forward *tim* primers: *5’-tggaataatcagaactttga-3’* (*ls-tim*); *5’-tggaataatcagaactttat-3’* (*s-tim*). Reverse (common) primer: *5’-agattccacaagatcgtgtt-3’* (*tim*).

### *cpo* sequencing

For *cpo* genotyping, the genomic DNA of several individuals from each strain used in the diapause assay was extracted, and the *cpo* region encompassing the nucleotide polymorphisms encoding the *cpo*^*A347V*^ and *cpo*^*48034(A/T)*^ was amplified via PCR. Primers used were: forward primer *5'-aacatccgttgctgctgtc-3’* reverse primer *5'-ccccaagctgtcacttttgt-3’* Templates were purified through minicolumns (Wizard® SV Gel and PCR Clean-Up System) and subsequently sequenced.

### Luciferase Reporter Gene Assay

The *FoxO*.*RE-Luciferase* transgene contains a firefly luciferase reporter gene under the control of 8 consecutive FoxO-response elements (FRE) [41]. *FoxO*.*RE-Luc* larvae were reared as for the diapause assay. Females were dissected in dry ice and, then, frozen at -80°C until processing. Luciferase extraction was performed using the Promega Luciferase Assay System (Firefly Luciferase). 150μL lysis buffer (LB) was added to 40 abdomens (9 biological replicates). Samples were frozen in liquid nitrogen and thawed in a 37°C water bath three times and then centrifuged to remove debris. This process was repeated and the two resulting supernatants were combined and stored at -70°C. Luciferase activity of adult protein extracts was measured using a Berthold Technologies Luminometer microplate scintillation and counter. 100μL of Promega Luciferase Assay Reagent was added to 20μL of protein extract and light production was measured in relative light units (RLU) emitted over a 10 s time period. Final Luciferase values (Lv) were normalized to the protein concentration (RLU/μg of protein). Protein concentration was determined using the Pierce BCA Protein Assay Kit–Reducing Agent Compatible. Data from two temperatures (12 and 23°C) were compared using t-test.

### qPCR and Gene expression

mRNA was extracted from 50 isolated heads (3 biological replicates) or 30 whole bodies (5 biological replicates) of adult females by using TRIzol (Invitrogen) and RNeasy Mini Kit and RNase-Free DNase Set (QIAGEN), respectively. The first-strand cDNA was synthesized by using the Invitrogen SuperScript II First-Strand Synthesis SuperMix. qPCR was performed in LightCycler DNA Master SYBR Green I (Roche) on LightCycler 480 System (Roche). Primers are following: *dilp2* (heads), F: *5’-gtatggtgtgcgaggagtat*, R: *5’-tgagtacacccccaagatag*; *dilp5* (heads), F: *5’-agttctcctgttcctgatcc*, R: *5’-cagtgagttcatgtggtgag*; *rp49* (heads), F: *5’-agggtatcgacaacagagtg*, R: *5’-caccaggaacttcttgaatc*; *dilp2* (body), F: *5’-acgaggtgctgagtatggtgtgcg*, R: *5’-cacttcgcagcggttccgatatcg; dilp5* (body), F: *5’-tgttcgccaaacgaggcaccttgg*, R: *5’-cacgatttgcggcaacaggagtcg*; *rpL23* (body), F: *5’-gacaacaccggagccaagaacc*, R: *5’-gtttgcgctgccgaataaccac*. For each *dilps* transcript, we normalized message levels relative to *rpL23* (whole body) or *rp49* (isolated heads) housekeeping genes by using the 2^-ΔΔCT^. Data were analyzed using t-test.

## Supporting Information

S1 Fig Ovarian development in highly diapausing lines at 23°C is normal and comparable to controls. Females from all highly diapausing lines used throughout the experiments (*Df(3L)dilp1-5/dilp2*,*3*,*5*^*-*^; *chico*^*KG00032*^; *dilp2>hid*,*rpr*; *InsP3>hid*,*rpr*; *dilp2(p)>Ork1* and *c929>sImp-L2*) exposed for 11 days at 23°C exhibited normal gonadal maturation comparable to controls (*c929>+*; *InsP3>+*; *dilp2>+* and *dilp2(p)>+*). Therefore, at 23°C all ovaries were vitellogenic, indicating that the mutants listed above and genetically manipulated strains were fertile. Consequently, the non-vitellogenic phenotypes observed in diapausing conditions (12°C) were true diapause phenotypes. Bars = 0.2 mm.
(TIF)

S1 Table LD conditions, and cpoA347V, cpo SNP 48034 and tim genetic backgrounds considered throughout the experiments. (DOCX)

S2 Table Highly diapausing genotypes are all fertile. Females of high diapause strains dissected after 11 days at 23°C show no diapause. Flies were collected after 5 h post eclosion and exposed to 23°C LD12.12 for 11 days before dissection.
(DOCX)
