# Supplementary figures and images for: The Insulin-Like Proteins dILPs-2/5 Determine Diapause Inducibility in Drosophila
Source: PLoS One. 2016 Sep 30;11(9):e0163680. doi: 10.1371/journal.pone.0163680 (PMC5045170; doi:10.1371/journal.pone.0163680)

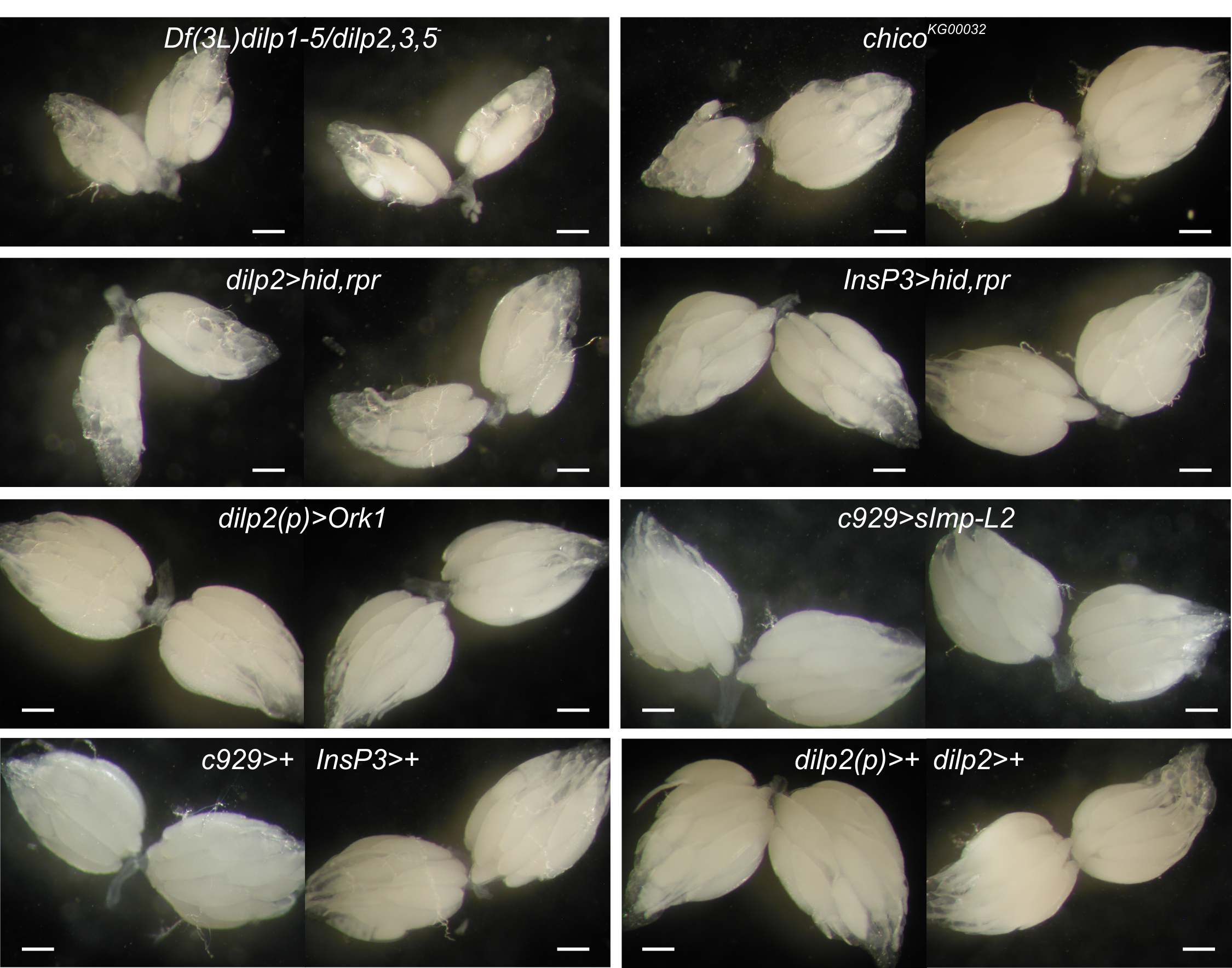

Supplement: S1 Fig — Females from all highly diapausing lines used throughout the experiments (Df(3L)dilp1-5/dilp2,3,5-; chicoKG00032; dilp2>hid,rpr; InsP3>hid,rpr; dilp2(p)>Ork1 and c929>sImp-L2) exposed for 11 days at 23°C exhibited normal gonadal maturation comparable to controls (c929>+; InsP3>+; dilp2>+ and dilp2(p)>+). Therefore, at 23°C all ovaries were vitellogenic, indicating that the mutants listed above and genetically manipulated strains were fertile. Consequently, the non-vitellogenic phenotypes observed in diapausing conditions (12°C) were true diapause phenotypes. Bars = 0.2 mm. (TIF) [file pone.0163680.s001.tif]
