# Supplementary material for: The Insulin-Like Proteins dILPs-2/5 Determine Diapause Inducibility in Drosophila
Source: PLoS One. 2016 Sep 30;11(9):e0163680. doi: 10.1371/journal.pone.0163680 (PMC5045170; doi:10.1371/journal.pone.0163680)
Supplement: S1 Table — (DOCX) [file pone.0163680.s002.docx]

**S1 Table. Summary table about all diapause results with respective LD conditions, *cpo^A347V^*, *cpo* SNP 48034 and *tim* backgrounds**

| Genotype and condition (when not specified 12°C for 11 days) | LD | % diapause±SD | *cpo^A347V^* | *cpo* SNP 48034 (A or T) | *tim* |
| --- | --- | --- | --- | --- | --- |
| *dilp2>+* | 16:8 | 34.9±6.5 | V/A | *A/A* | *s/s* |
| *dilp2>hid,rpr* | 16:8 | 97.6±2.9 | *V/A* | *A/T* | *s/s* |
| *+>hid,rpr* | 16:8 | 31.8±5.2 | *A/A* | *A/T* | *s/s* |
| *InsP3>hid,rpr* | 16:8 | 97.3±1.7 | *A/A* | *A/T* | *s/s* |
| *InsP3>+* | 16:8 | 40.7±2.5 | *A/A* | *A/A* | *s/s* |
| *dilp2(p)>+* | 8:16 | 42.7±1.6 | *A/A* | *A/A* | *s/s* |
| *dilp2(p)>NaChBac* | 8:16 | 8.7±3.5 | A/A | *A/A* | *s/s* |
| *dilp2>+* | 8:16 | 40.3±3.7 | V/A | *A/A* | *s/s* |
| *dilp2>NaChBac* | 8:16 | 0.8±1.0 | V/A | *A/A* | *s/s* |
| *+>NaChBac* | 8:16 | 45.3±2.6 | A/A | *A/A* | *s/s* |
| *dilp2(p)>Ork1* | 8:16 | 91.9±2.8 | A/A | *A/A* | *s/s* |
| *dilp2(p)>NOrk1* | 8:16 | 36.8±5.0 | A/A | *A/A* | *s/s* |
| *+>Ork1* | 8:16 | 36.3±4.7 | A/A | *A/A* | *s/s* |
| *+>NOrk1* | 8:16 | 36.8±4.2 | *A/A* | *A/A* | *s/s* |
| *white^1118^* | 16:8 | 36.8±4.0 | *A/A* | *A/A* | *s/s* |
| *Df(3L)dilp1-5^-/-^* | 16:8 | 100.0±0.0 | *V/V* | *A/A* | *s/s* |
| *Df(3L)dilp1-5^+/-^* | 16:8 | 38.1±1.9 | *A/V* | *A/A* | *s/s* |
| *dilp2,3,5^-/-^* | 16:8 | 100.0±0.0 | *V/V* | *A/A* | *s/s* |
| *dilp2,3,5^+/-^* | 16:8 | 37.5±4.2 | *A/V* | *A/A* | *s/s* |
| *Df(3L)/dilp2,3,5^-^* | 16:8 | 100.0±0.0 | *V/V* | *A/A* | *s/s* |
| *dilp2^-/-^* | 16:8 | 59.8±7.0 | *V/V* | *deletion* | *ls/ls* |
| *dilp3^-/-^* | 16:8 | 45.4±3.8 | *V/V* | *A/A* | *ls/ls* |
| *dilp5^-/-^* | 16:8 | 57.5±3.7 | *V/A* | *A/A* | *ls/ls* |
| *Control ls-tim* | 16:8 | 44.3±4.2 | *A/A* | *A/A* | *ls/ls* |
| *chico^KG00032^* | 16:8 | 88.5±3.7 | *A/A* | *A/A* | *ls/ls* |
| *Control y^-/-^* | 16:8 | 22.7±4.3 | *A/A* | *A/A* | *s/s* |
| *y^-/-^;;InR^EY00681^* | 16:8 | 90.3±6.2 | *A/A* | *A/A* | *s/s* |
| *c929>+* | 16:8 | 37.2±4.5 | *V/A* | *A/A* | *s/s* |
| *c929>sImp-L2* | 16:8 | 100.0±0.0 | *V/A* | *A/A* | *s/s-ls* |
| *+>sImp-L2* | 16:8 | 22.2±13.4 | *A/A* | *A/A* | *s/s-ls* |
| *dilp2(p)>+* | 8:16 | 46.2±5.6 | *A/A* | *A/A* | *s/s* |
| *dilp2(p)>dilp2* | 8:16 | 6.0±2.4 | *A/A* | *A/A* | *s/s* |
| *dilp2(p)>dilp5* | 8:16 | 9.1±2.0 | *A/A* | *A/A* | *s/s* |
| *dilp2>+* | 8:16 | 41.3±6.3 | *V/A* | *A/A* | *s/s* |
| *dilp2>dilp2* | 8:16 | 5.6±2.5 | *V/A* | *A/A* | *s/s* |
| *dilp2>dilp5* | 8:16 | 7.4±4.7 | *V/A* | *A/A* | *s/s* |
| *+>dilp2* | 8:16 | 54.4±11.0 | *A/A* | *A/A* | *s/s* |
| *+>dilp5* | 8:16 | 42.9±4.9 | *A/A* | *A/A* | *s/s* |
| *c929>+* | 8:16 | 41.9±4.1 | *V/A* | *A/A* | *s/s* |
| *c929>dilp2* | 8:16 | 0.7±0.7 | *V/A* | *A/A* | *s/s* |
| *c929>dilp5* | 8:16 | 10.1±3.2 | *V/A* | *A/A* | *s/s* |
| *akh>+* | 8:16 | 47.0±2.3 | *A/A* | *A/A* | *ls/s* |
| *akh>dilp2* | 8:16 | 3.7±3.5 | *A/A* | *A/A* | *ls/s* |
| *akh>dilp5* | 8:16 | 5.9±2.2 | *A/A* | *A/A* | *ls/s* |
| *+>dilp2* | 8:16 | 47.2±2.8 | *A/A* | *A/A* | *ls/s* |
| *+>dilp5* | 8:16 | 45.1±1.3 | *A/A* | *A/A* | *ls/s* |
| *hmgcr^Di-11^>+* | 8:16 | 59.1±4.0 | *V/A* | *A/A* | *s/s* |
| *hmgcr^Di-11^>dilp2* | 8:16 | 2.1±1.2 | *V/A* | *A/A* | *s/s* |
| *+>dilp2* | 8:16 | 54.4±11.0 | *A/A* | *A/A* | *s/s* |
| *Lk6^DJ634^>+* | 8:16 | 60.2±9.8 | *A/A* | *A/A* | *ls/s* |
| *Lk6^DJ634^>dilp2* | 8:16 | 1.7±1.3 | *A/A* | *A/A* | *ls/s* |
| *cg>+* | 8:16 | 37.6±5.2 | *A/A* | *A/A* | *s/s* |
| *cg>dilp5* | 8:16 | 1.4±1.0 | *A/A* | *A/A* | *s/s* |
| *dilp2>+* | 8:16 | 40.3±3.7 | *V/A* | *A/A* | *s/s* |
| *dilp2>+* | 16:8 | 33.2±4.6 | *V/A* | *A/A* | *s/s* |
| *Foxo-luc* | 8:16 | 49.5±4.8 | *V/V* | *T/A* | *s/s* |
| *Foxo-luc* | 16:8 | 36.2±3.4 | *V/V* | *T/A* | *s/s* |
| *dilp2>+* | 16:8 | 34.9±3.1 | *V/A* | *A/A* | *s/ls* |
| *dilp2>dilp2-RNAi* | 16:8 | 65.5±8.1 | *V/A* | *A/A* | *s/ls* |
| *dilp2>dilp5-RNAi* | 16:8 | 74.2±7.4 | *V/A* | *A/A* | *s/ls* |
| *+>dilp2-RNAi* | 16:8 | 47.7±3.2 | *A/A* | *A/A* | *s/ls* |
| *+>dilp5-RNAi* | 16:8 | 49.5±5.3 | *A/A* | *A/A* | *s/ls* |
| *white^1118^ 11 days at 12°C + 5 at 12°C* | 16:8 | 32.2±3.4 | *A/A* | *A/A* | *s/s* |
| *Df(3L)dilp1-5^-/-^ 11 days at 12°C + 5 at 12°C* | 16:8 | 100.0±0.0 | *V/V* | *A/A* | *s/s* |
| *Df(3L)/dilp2,3,5^-^ 11 days at 12°C + 5 at 12°C* | 16:8 | 100.0±0.0 | *V/V* | *A/A* | *s/s* |
| *dilp2(p)>+ 11 days at 12°C + 5 at 12°C* | 16:8 | 28.8±3.5 | *A/A* | *A/A* | *s/s* |
| *white^1118^ 11 days at 12°C (16:8) + 5 at 15°C* | 16:8 | 6.4±3.8 | *A/A* | *A/A* | *s/s* |
| *Df(3L)dilp1-5^-/-^ 11 days at 12°C (16:8) + 5 at 15°C* | 16:8 | 100.0±0.0 | *V/V* | *A/A* | *s/s* |
| *Df(3L)/dilp2,3,5^-^ 11 days at 12°C (16:8) + 5 at 15°C* | 16:8 | 100.0±0.0 | *V/V* | *A/A* | *s/s* |
| *dilp2(p)>+ 11 days at 12°C (16:8) + 5 at 15°C* | 16:8 | 6.0±1.3 | *A/A* | *A/A* | *s/s* |
| *white^1118^ 11 days at 12°C (16:8) + 5 at 19°C* | 16:8 | 0.6±0.8 | *A/A* | *A/A* | *s/s* |
| *Df(3L)dilp1-5^-/-^ 11 days at 12°C (16:8) + 5 at 19°C* | 16:8 | 76.4±2.4 | *V/V* | *A/A* | *s/s* |
| *Df(3L)/dilp2,3,5^-^ 11 days at 12°C (16:8) + 5 at 19°C* | 16:8 | 87.0±11.2 | *V/V* | *A/A* | *s/s* |
| *dilp2(p)>+ 11 days at 12°C (16:8) + 5 at 19°C* | 16:8 | 0.0±0.0 | *A/A* | *A/A* | *s/s* |
| *white^1118^ 11 days at 12°C (16:8) + 5 at 22°C* | 12:12 | 0.0±0.0 | *A/A* | *A/A* | *s/s* |
| *Df(3L)dilp1-5^-/-^* | 12:12 | 0.0±0.0 | *V/V* | *A/A* | *s/s* |
| *Df(3L)/dilp2,3,5^-^* | 12:12 | 0.0±0.0 | *V/V* | *A/A* | *s/s* |
| *dilp2(p)>+* | 12:12 | 0.0±0.0 | *A/A* | *A/A* | *s/s* |
| *dilp2>+ (28 days)* | 16:8 | 1.3±2.8 | V/A | *A/A* | *s/s* |
| *dilp2>hid,rpr (28 days)* | 16:8 | 66.0±12.0 | *V/A* | *A/T* | *s/s* |
| *+>hid,rpr (28 days)* | 16:8 | 0.0±0.0 | *A/A* | *A/T* | *s/s* |
| *InsP3>hid,rpr (28 days)* | 16:8 | 49.9±12.4 | *A/A* | *A/T* | *s/s* |
| *InsP3>+ (28 days)* | 16:8 | 18.4±5.5 | *A/A* | *A/A* | *s/s* |
| *dilp2(p)>+ (28 days)* | 16:8 | 0.0±0.0 | *A/A* | *A/A* | *s/s* |
| *dilp2(p)>Ork1 (28 days)* | 16:8 | 32.0±9.9 | A/A | *A/A* | *s/s* |
| *+>Ork1 (28 days)* | 16:8 | 0.0±0.0 | A/A | *A/A* | *s/s* |
| *white^1118^ (28 days)* | 16:8 | 13.8±7.4 | *A/A* | *A/A* | *s/s* |
| *Df(3L)dilp1-5^-/-^ (28 days)* | 16:8 | 100.0±0.0 | *V/V* | *A/A* | *s/s* |
| *Df(3L)/dilp2,3,5^-^ (28 days)* | 16:8 | 84.1±7.1 | *V/V* | *A/A* | *s/s* |
| *chico^KG00032^ (28 days)* | 16:8 | 45.8±9.1 | *A/A* | *A/A* | *ls/ls* |
| *y^-/-^;;InR^EY00681^ (28 days)* | 16:8 | 32.3±5.9 | *A/A* | *A/A* | *s/s* |
