# Supplementary material for: The Insulin-Like Proteins dILPs-2/5 Determine Diapause Inducibility in Drosophila
Source: PLoS One. 2016 Sep 30;11(9):e0163680. doi: 10.1371/journal.pone.0163680 (PMC5045170; doi:10.1371/journal.pone.0163680)
Supplement: S2 Table — Females of high diapause strains dissected after 11 days at 23°C show no diapause. Flies were collected after 5 h post eclosion and exposed to 23°C LD12.12 for 11 days before dissection. (DOCX) [file pone.0163680.s003.docx]

**S2 Table. Highly diapausing genotypes are all fertile.**

| Genotype | Vitellogenic flies | Non vitellogenic flies |
| --- | --- | --- |
| *dilp2>hid,rpr* | 35 | 0 |
| *InsP3>hid,rpr* | 38 | 0 |
| *dilp2(p)>Ork1* | 35 | 0 |
| *Df(3L)/Dilp2,3,5^-/-^* | 34 | 0 |
| *chico^KG00032^* | 27 | 0 |
| *y^-/-^;;InR^EY00681^* | 30 | 0 |
| *c929>sImp-L2* | 33 | 0 |

**S2 Table.** Females of high diapause strains dissected after 11 days at 23°C show no diapause. Flies were collected after 5 h post eclosion and exposed to 23°C LD12.12 for 11 days before dissection.
